# Supplementary material for: Healthy behaviors at age 50 years and frailty at older ages in a 20-year follow-up of the UK Whitehall II cohort: A longitudinal study
Source: PLoS Med. 2020 Jul 6;17(7):e1003147. doi: 10.1371/journal.pmed.1003147 (PMC7337284; doi:10.1371/journal.pmed.1003147)
Supplement: S1 Table — (DOCX) [file pmed.1003147.s001.docx]

**S1 Table. Wave of extraction of health behaviors at age 50**

|  | **Data collection wave** | | | |
| --- | --- | --- | --- | --- |
|  | **1985** | **1991** | **1997** | **2002** |
| **N participants with data at age 50 extracted** | 2093 | 1751 | 2046 | 467 |
| **Follow-up, Mean (SD), y** | 25.0 (4.3) | 21.5 (3.8) | 16.3 (3.4) | 11.5 (2.2) |
| **Follow-up range, y** | 14.9 to 31.1 | 9.4 to 25.1 | 4.1 to 19.3 | 3.9 to 13.7 |
