# Supplementary material for: Healthy behaviors at age 50 years and frailty at older ages in a 20-year follow-up of the UK Whitehall II cohort: A longitudinal study
Source: PLoS Med. 2020 Jul 6;17(7):e1003147. doi: 10.1371/journal.pmed.1003147 (PMC7337284; doi:10.1371/journal.pmed.1003147)
Supplement: S2 Table — (DOCX) [file pmed.1003147.s002.docx]

**S2 Table. Chronic conditions at age 50 according to frailty status at the end of follow-up***

| **Chronic conditions at age 50** | **Non-frail**  **N=5912** | **Frail**  **N=445** | **p** |
| --- | --- | --- | --- |
| Obesity | 593 (10.0) | 84 (18.9) | <0.001 |
| Hypertension | 1225 (20.7) | 108 (24.3) | 0.08 |
| Diabetes | 106 (1.8) | 12 (2.7) | 0.17 |
| Depression | 139 (2.3) | 22 (4.9) | 0.001 |
| Arthritis | 3 (0.1) | 0 (0.0) | 0.64 |
| Chronic obstructive Pulmonary disease | 1 (0.0) | 0 (0.0) | 0.78 |
| Coronary heart disease | 137 (2.3) | 14 (3.1) | 0.27 |
| Stroke | 4 (0.1) | 1 (0.2) | 0.25 |
| Cancer | 32 (0.5) | 2 (0.4) | 0.78 |
