# Supplementary material for: Healthy behaviors at age 50 years and frailty at older ages in a 20-year follow-up of the UK Whitehall II cohort: A longitudinal study
Source: PLoS Med. 2020 Jul 6;17(7):e1003147. doi: 10.1371/journal.pmed.1003147 (PMC7337284; doi:10.1371/journal.pmed.1003147)
Supplement: S3 Table — (DOCX) [file pmed.1003147.s003.docx]

**S3 Table. Characteristics of the study sample according to the number of healthy behaviors at age 50***

| **Characteristics** | **Number of healthy behaviors at age 50** | | | | | **p trend** |
| --- | --- | --- | --- | --- | --- | --- |
|  | **0** | **1** | **2** | **3** | **4** |  |
|  | **N=142** | **N=1002** | **N=2342** | **N=2167** | **N=704** |  |
| Sex |  |  |  |  |  | 0.24 |
| Men | 94 (66.2) | 660 (65.8) | 1685 (71.9) | 1615 (74.5) | 447 (63.5) |  |
| Women | 48 (33.8) | 342 (34.1) | 657 (28.0) | 552 (25.5) | 257 (36.5) |  |
| Ethnicity |  |  |  |  |  | <0.001 |
| Caucasian | 126 (88.7) | 840 (83.8) | 2132 (91.0) | 2046 (94.4) | 686 (97.4) |  |
| Non-Caucasian | 16 (11.3) | 162 (16.2) | 210 (9.0) | 121 (5.6) | 18 (2.6) |  |
| Marital status |  |  |  |  |  | <0.001 |
| Married/cohabiting | 99 (69.7) | 716 (71.5) | 1806 (77.1) | 1749 (80.7) | 572 (81.2) |  |
| Single, divorced or. widowed | 43 (30.3) | 286 (28.5) | 536 (22.9) | 418 (19.3) | 132 (18.7) |  |
| Education |  |  |  |  |  | <0.001 |
| No academic. qualification | 29 (20.4) | 138 (13.8) | 268 (11.4) | 176 (8.2) | 52 (7.4) |  |
| High school | 59 (41.5) | 380 (37.9) | 742 (31.7) | 699 (32.3) | 182 (25.8) |  |
| Higher secondary | 26 (18.3) | 265 (26.5) | 665 (28.4) | 576 (26.6) | 205 (29.1) |  |
| University | 21 (14.8) | 175 (17.5) | 483 (20.6) | 542 (25.0) | 190 (27.0) |  |
| Higher university degree | 7 (4.9) | 44 (4.4) | 184 (7.9) | 174 (8.0) | 75 (10.6) |  |
| Occupational position |  |  |  |  |  | <0.001 |
| Low | 32 (22.5) | 235 (23.4) | 326 (13.9) | 188 (8.7) | 64 (9.1) |  |
| Intermediate | 78 (54.9) | 444 (44.3) | 1048 (44.7) | 970 (44.8) | 284 (40.3) |  |
| High | 32 (22.5) | 323 (32.2) | 968 (41.3) | 1009 (46.6) | 356 (50.6) |  |
| Number of chronic conditions |  |  |  |  |  | 0.05 |
| 0 | 89 (62.7) | 670 (66.9) | 1616 (69.0) | 1501 (69.3) | 494 (70.2) |  |
| 1 | 38 (26.8) | 257 (25.6) | 568 (24.3) | 524 (24.2) | 162 (23.0) |  |
| 2 or more | 15 (10.6) | 75 (7.5) | 158 (6.7) | 142 (6.6) | 48 (6.8) |  |

*Values are numbers (percentages). Percentages are reported in column.
