# Supplementary material for: Healthy behaviors at age 50 years and frailty at older ages in a 20-year follow-up of the UK Whitehall II cohort: A longitudinal study
Source: PLoS Med. 2020 Jul 6;17(7):e1003147. doi: 10.1371/journal.pmed.1003147 (PMC7337284; doi:10.1371/journal.pmed.1003147)
Supplement: S4 Table — CI, confidence interval; HR, hazard ratio. (DOCX) [file pmed.1003147.s004.docx]

**S4 Table. Association between the number of healthy behaviors at age 50 and the risk of frailty over a 20-year follow-up**

| **Number of healthy behaviors at age 50** |  |  | |  | |  | | **Model 1*** | | | |  | | **Model 2^†^** | | | | | |  | **Model 3**^‡^ | | | |
| --- | --- | --- | --- | --- | --- | --- | --- | --- | --- | --- | --- | --- | --- | --- | --- | --- | --- | --- | --- | --- | --- | --- | --- | --- |
|  | **N frail /**  **N total** |  | |  | | **Frail %** | | **HR (95%CI)** | | **p** | |  | | **HR (95%CI)** | | **p** | |  | | **HR (95%CI)** | | | **p** | |
|  |  |  | |  | |  | |  | |  | |  | |  | |  | |  | |  | | |  | |
| 0 | 20/142 |  | |  | | 14.08 | | 1 (ref) | |  | |  | | 1 (ref) | |  | |  | | 1 (ref) | | |  | |
| 1 | 127/1002 |  | |  | | 12.67 | | 0.68 (0.41 to 1.11) | | 0.12 | |  | | 0.69 (0.42 to 1.12) | | 0.13 | |  | | 0.74 (0.45 to 1.21) | | | 0.22 | |
| 2 | 177/2342 |  | |  | | 7.56 | | 0.41 (0.25 to 0.67) | | 0.001 | |  | | 0.44 (0.27 to 0.71) | | 0.001 | |  | | 0.47 (0.29 to 0.77) | | | 0.01 | |
| 3 | 96/2167 |  | |  | | 4.43 | | 0.26 (0.16 to 0.44) | | <0.001 | |  | | 0.29 (0.18 to 0.49) | | <0.001 | |  | | 0.32 (0.19 to 0.53) | | | <0.001 | |
| 4 | 25/704 |  | |  | | 3.55 | | 0.23 (0.13 to 0.43) | | <0.001 | |  | | 0.26 (0.14 to 0.48) | | <0.001 | |  | | 0.28 (0.15 to 0.52) | | | <0.001 | |
|  |  |  | |  | |  | |  | |  | |  | |  | |  | |  | |  | | |  | |
| Per one additional healthy behavior | |  |  | |  | | 0.65 (0.59 to 0.73) | | <0.001 | |  | | 0.68 (0.61 to 0.76) | | <0.001 | |  | | 0.69 (0.62 to 0.76) | | | <0.001 | |  |

*****Model 1: age as timescale, adjusted for sex, ethnicity, marital status, and wave of inclusion.

**^†^** Model 2: model 1 additionally adjusted for education and occupational position.

^‡^ Model 3: model 2 additionally adjusted for the number of morbidities at age 50.

CI: confidence interval, HR: Hazard Ratio
