# Supplementary material for: Healthy behaviors at age 50 years and frailty at older ages in a 20-year follow-up of the UK Whitehall II cohort: A longitudinal study
Source: PLoS Med. 2020 Jul 6;17(7):e1003147. doi: 10.1371/journal.pmed.1003147 (PMC7337284; doi:10.1371/journal.pmed.1003147)
Supplement: S5 Table — CI, confidence interval; HR, hazard ratio. (DOCX) [file pmed.1003147.s005.docx]

**S5 Table. Association between the number of healthy behaviors at age 50 and risk of frailty using Cox regression only and with inverse probability weighting**

| **Number of healthy behaviors at age 50** | **N frail /**  **N total** |  |  | **Cox regression*** | |  | **Weighted Cox regression^†^** | |
| --- | --- | --- | --- | --- | --- | --- | --- | --- |
|  |  | **Frail %** |  | **HR (95%CI)** | **p** |  | **HR (95%CI)** | **p** |
|  |  |  |  |  |  |  |  |  |
| 0 | 20/142 | 14.08 |  | 1 (ref) |  |  | 1 (ref) |  |
| 1 | 127/1002 | 12.67 |  | 0.75 (0.45 to 1.20) | 0.25 |  | 0.72 (0.44 to 1.15) | 0.15 |
| 2 | 177/2342 | 7.56 |  | 0.49 (0.31 to 0.79) | <0.01 |  | 0.51 (0.32 to 0.82) | <0.01 |
| 3 | 96/2167 | 4.43 |  | 0.31 (0.19 to 0.52) | <0.001 |  | 0.33 (0.20 to 0.54) | <0.001 |
| 4 | 25/704 | 3.55 |  | 0.29 (0.16 to 0.54) | <0.001 |  | 0.28 (0.15 to 0.51) | <0.001 |
|  |  |  |  |  |  |  |  |  |
| Per one additional healthy behavior | |  |  | 0.68 (0.62 to 0.76) | <0.001 |  | 0.70 (0.63 to 0.78) | <0.001 |

* Cox regression with age as a timescale and adjusted for sex, ethnicity, marital status and wave of inclusion, education, occupational position, and number of morbidities at age 50.

^†^ Cox regression using inverse probability weighting, with age as a timescale and adjusted for sex, ethnicity, marital status and wave of inclusion, education, occupational position, and number of morbidities at age 50.

CI: confidence interval, HR: Hazard Ratio
