# Supplementary material for: Healthy behaviors at age 50 years and frailty at older ages in a 20-year follow-up of the UK Whitehall II cohort: A longitudinal study
Source: PLoS Med. 2020 Jul 6;17(7):e1003147. doi: 10.1371/journal.pmed.1003147 (PMC7337284; doi:10.1371/journal.pmed.1003147)
Supplement: S6 Table — CI, confidence interval; HR, hazard ratio. (DOCX) [file pmed.1003147.s006.docx]

**S6 Table. Association between different definitions of healthy alcohol consumption at age 50 and onset of frailty over a mean follow-up of 20 years**

|  | |  |  |  | **Model 1*^†^** | |  | **Model 2***^‡^ | |  | **Model 3***^§^ | |  | **Model 4**^‖^ | |
| --- | --- | --- | --- | --- | --- | --- | --- | --- | --- | --- | --- | --- | --- | --- | --- |
| **Healthy alcohol consumption** | | **N frail /**  **N total** | **Frail %** |  | **HR (95%CI)** | **p** |  | **HR (95%CI)** | **p** |  | **HR (95%CI)** | **p** |  | **HR (95%CI)** | **p** |
| *Moderate alcohol consumption* | | |  |  |  |  |  |  |  |  |  |  |  |  |  |
| No | | 220/2814 | 7.82 |  | 1 (ref) |  |  | 1 (ref) |  |  | 1 (ref) |  |  | 1 (ref) |  |
| Yes | | 225/3543 | 6.35 |  | 0.68 (0.56 to 0.82) | <0.001 |  | 0.69 (0.57 to 0.84) | <0.001 |  | 0.71 (0.59 to 0.86) | 0.001 |  | 0.73 (0.61 to 0.88) | <0.001 |
| *No alcohol consumption* | | |  |  |  |  |  |  |  |  |  |  |  |  |  |
| No | | 329/5368 | 6.13 |  | 1 (ref) |  |  | 1 (ref) |  |  | 1 (ref) |  |  | 1 (ref) |  |
| Yes | | 116/989 | 11.73 |  | 1.55 (1.24 to 1.95) | <0.001 |  | 1.38 (1.10 to 1.72) | 0.005 |  | 1.42 (1.13 to 1.78) | 0.003 |  | 1.36 (1.08 to 1.70) | 0.008 |
|  | *No or moderate alcohol consumption* | | | | |  |  |  |  |  |  |  |  |  |  |
| No | | 104/1825 | 5.70 |  | 1 (ref) |  |  | 1 (ref) |  |  | 1 (ref) |  |  | 1 (ref) |  |
| Yes | | 341/4532 | 7.52 |  | 0.83 (0.65 to 1.06) | 0.13 |  | 0.78 (0.62 to 1.00) | 0.05 |  | 0.81 (0.63 to 1.04) | 0.09 |  | 0.83 (0.66 to 1.06) | 0.14 |

**^†^**Model 1: age as a timescale, adjusted for sex, ethnicity, marital status, and wave of inclusion.

^‡^Model 2: model 1 additionally adjusted for education and occupational position.

^§^Model 3: model 2 additionally adjusted for the number of morbidities at age 50.

^‖^Model 4: model 3 additionally adjusted for all other healthy behaviors.

CI: confidence interval, HR: Hazard Ratio
