# Supplementary material for: Healthy behaviors at age 50 years and frailty at older ages in a 20-year follow-up of the UK Whitehall II cohort: A longitudinal study
Source: PLoS Med. 2020 Jul 6;17(7):e1003147. doi: 10.1371/journal.pmed.1003147 (PMC7337284; doi:10.1371/journal.pmed.1003147)
Supplement: S7 Table — CI, confidence interval; HR, hazard ratio. (DOCX) [file pmed.1003147.s007.docx]

|  |  |  |  | **Model 1*** | |  | **Model 2^†^** | |  | **Model 3**^‡^ | |  | **Model 4**^§^ | |
| --- | --- | --- | --- | --- | --- | --- | --- | --- | --- | --- | --- | --- | --- | --- |
|  | **N frail /**  **N total** |  | **Frail %** | **HR (95%CI)** | **p** |  | **HR (95%CI)** | **p** |  | **HR (95%CI)** | **p** |  | **HR (95%CI)** | **p** |
| *Sedentary time* |  |  |  |  |  |  |  |  |  |  |  |  |  |  |
| <2.8 hours/day | 112/1816 |  | 6.17 | 0.74 (0.57 to 0.96) | 0.02 |  | 0.73 (0.56 to 0.95) | 0.02 |  | 0.75 (0.57 to 0.97) | 0.03 |  | 0.74 (0.57 to 0.97) | 0.03 |
| 2.8-4.4 hours/day | 103/1801 |  | 5.72 | 0.77 (0.59 to 1.00) | 0.05 |  | 0.81 (0.62 to 1.06) | 0.12 |  | 0.83 (0.63 to 1.08) | 0.17 |  | 0.88 (0.67 to 1.15) | 0.34 |
| >4.4 hours/day | 136/1679 |  | 8.10 | 1 (ref) |  |  | 1 (ref) |  |  | 1 (ref) |  |  | 1 (ref) |  |
| *Healthy sedentary time* |  |  |  |  |  |  |  |  |  |  |  |  |  |  |
| <4.4 hours/day | 215/3617 |  | 5.94 | 0.75 (0.60 to 0.94) | 0.01 |  | 0.77 (0.61 to 0.96) | 0.02 |  | 0.78 (0.62 to 0.98) | 0.03 |  | 0.79 (0.63 to 0.99) | 0.04 |
| ≥4.4 hours/day | 136/1679 |  | 8.10 | 1 (ref) |  |  | 1 (ref) |  |  | 1 (ref) |  |  | 1 (ref) |  |
| *Number of healthy behaviors* |  |  |  |  |  |  |  |  |  |  |  |  |  |  |
| 0-1^‖^ | 19/167 |  | 11.38 | 1 (ref) |  |  | 1 (ref) |  |  | 1 (ref) |  |  | NA |  |
| 2 | 81/782 |  | 10.36 | 0.69 (0.42 to 1.14) | 0.15 |  | 0.71 (0.43 to 1.18) | 0.18 |  | 0.78 (0.47 to 1.30) | 0.34 |  | NA |  |
| 3 | 126/1847 |  | 6.82 | 0.44 (0.27 to 0.72) | 0.001 |  | 0.45 (0.27 to 0.74) | 0.002 |  | 0.51 (0.31 to 0.85) | 0.01 |  | NA |  |
| 4 | 89/1810 |  | 4.92 | 0.29 (0.17 to 0.48) | <0.001 |  | 0.29 (0.18 to 0.50) | <0.001 |  | 0.34 (0.20 to 0.58) | <0.001 |  | NA |  |
| 5 | 36/690 |  | 5.22 | 0.30 (0.17 to 0.53) | <0.001 |  | 0.31 (0.17 to 0.57) | <0.001 |  | 0.35 (0.19 to 0.64) | <0.001 |  | NA |  |
| Per one additional healthy behavior | | |  | 0.70 (0.63 to 0.78) | <0.001 |  | 0.71 (0.63 to 0.79) | <0.001 |  | 0.72 (0.65 to 0.81) | <0.001 |  | NA |  |

**S7 Table. Association between sedentary behaviors in 1997 (mean age=55.5 (SD=6.0) years) and onset of frailty over a mean follow-up of 16 years, N=5296**

*Model 1: age as a timescale, adjusted for sex, ethnicity, marital status.

**^†^**Model 2: model 1 additionally adjusted for education and occupational position.

^‡^Model 3: model 2 additionally adjusted for the number of morbidities in 1997.

^§^Model 4: model 3 additionally adjusted for all other healthy behaviors.

^‖^ The small number of participants with no healthy behaviors (N frail / N total =4/20) led us to combine those with no or 1 healthy behavior in the same category.

CI: confidence interval, HR: Hazard Ratio
