# Supplementary figures and images for: Healthy behaviors at age 50 years and frailty at older ages in a 20-year follow-up of the UK Whitehall II cohort: A longitudinal study
Source: PLoS Med. 2020 Jul 6;17(7):e1003147. doi: 10.1371/journal.pmed.1003147 (PMC7337284; doi:10.1371/journal.pmed.1003147)

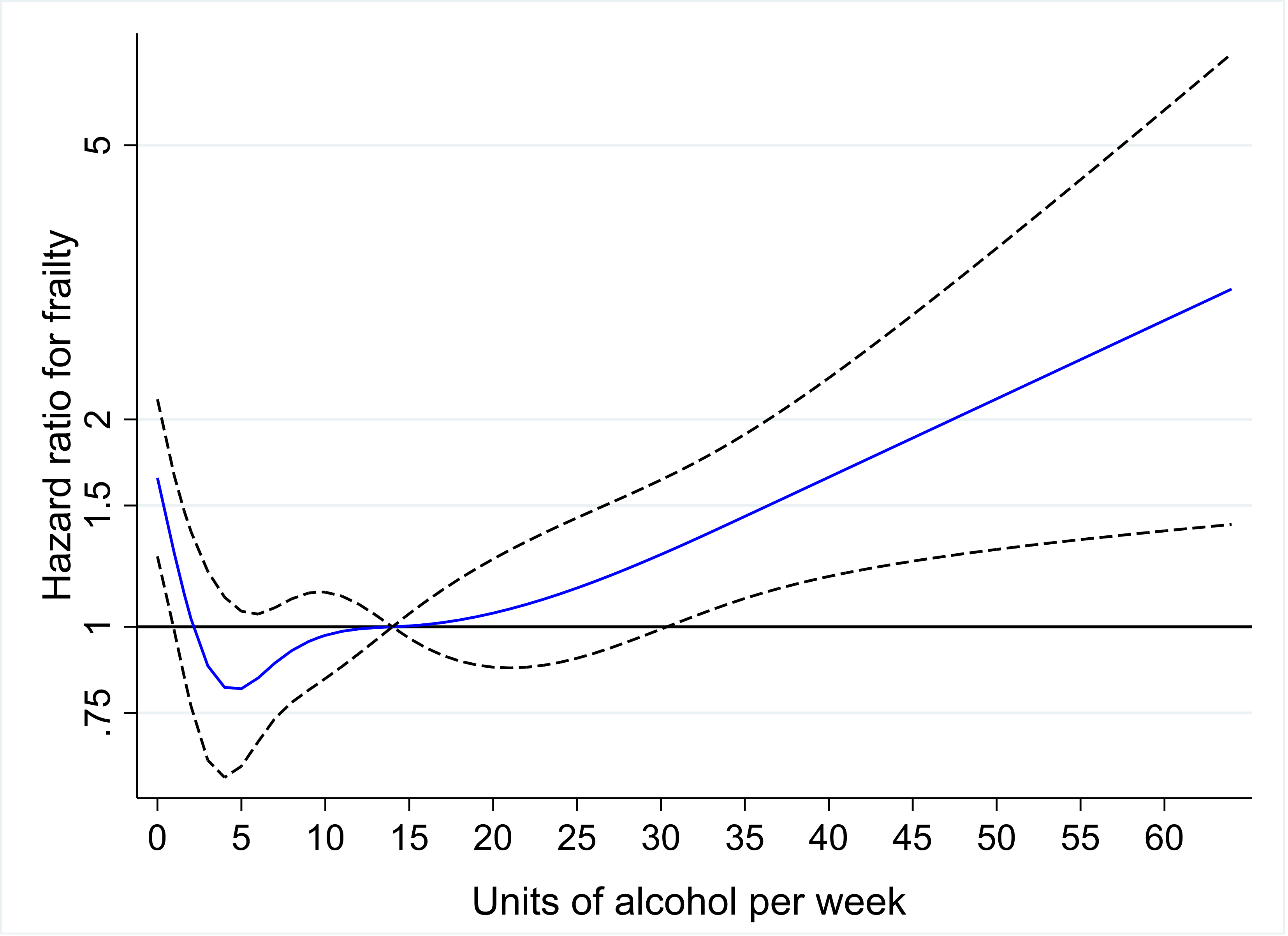

Supplement: S1 Fig — Blue line represents the hazard ratio for frailty compared with “14 units of alcohol per week” (reference), and black dashed line represents the corresponding 95% confidence interval estimated based on a Cox regression model with age at timescale adjusted for sex, ethnicity, marital status, and wave at inclusion. (TIF) [file pmed.1003147.s008.tif]

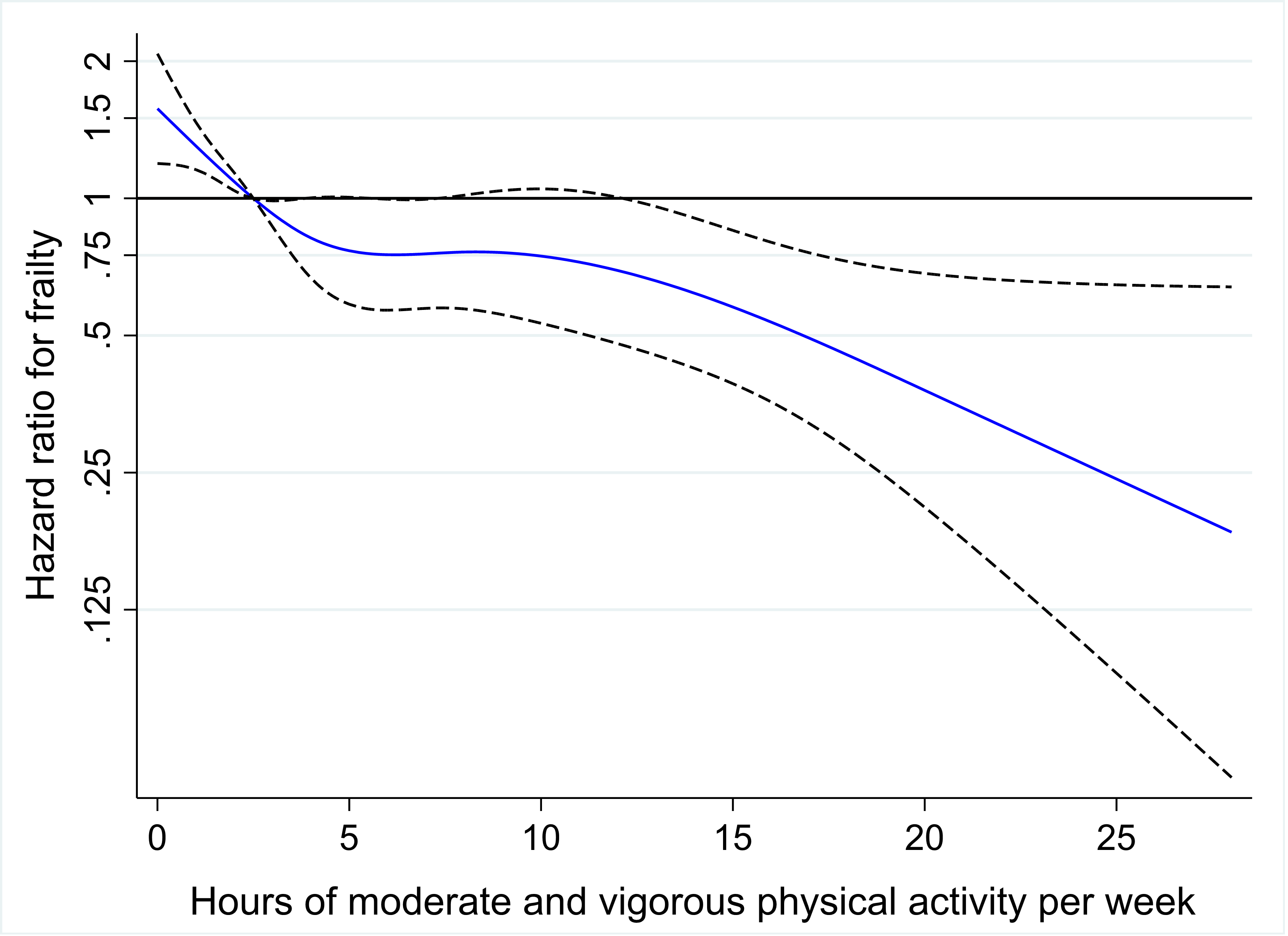

Supplement: S2 Fig — Blue line represents the hazard ratio for frailty compared with 2.5 hours of moderate and vigorous physical activity (reference), and black dashed line represents the corresponding 95% confidence interval estimated based on a Cox regression model with age at timescale adjusted for sex, ethnicity, marital status, and wave at inclusion. (TIF) [file pmed.1003147.s009.tif]

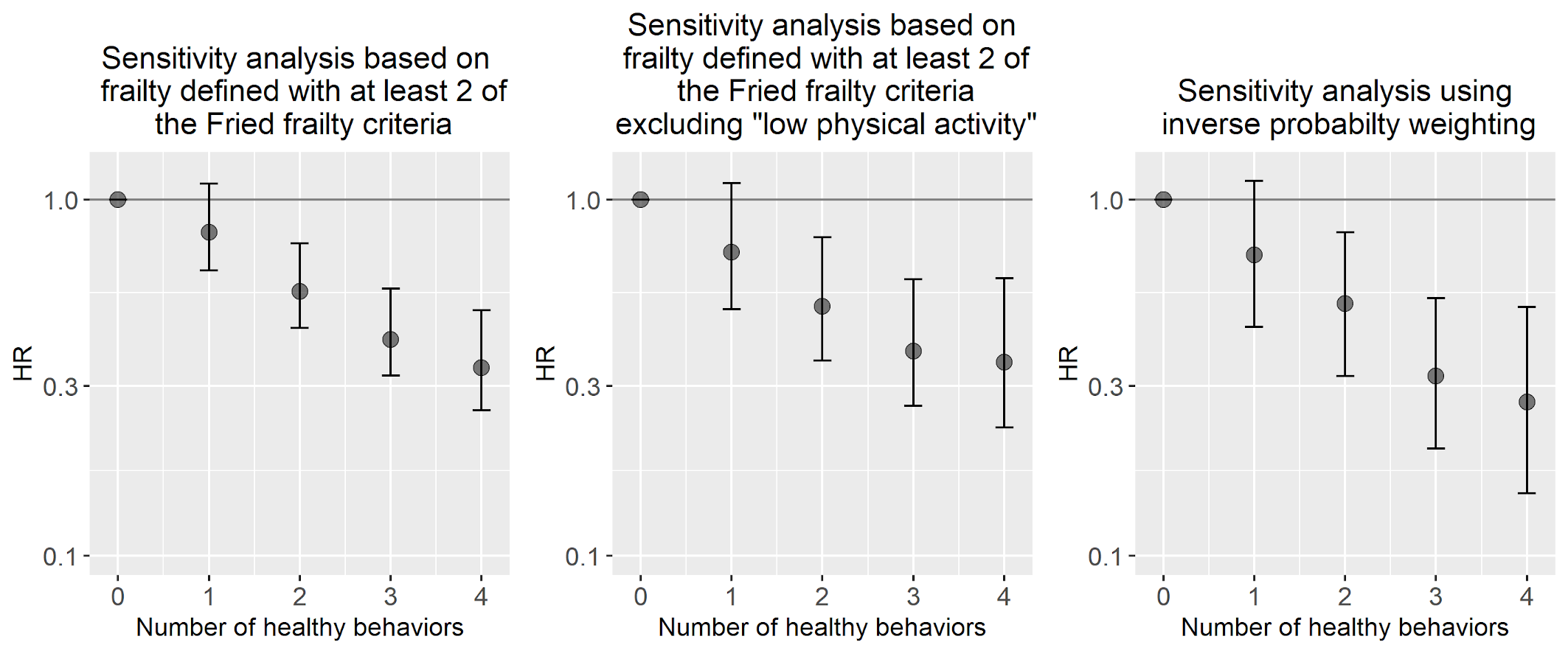

Supplement: S3 Fig — Age was used as timescale, and models are adjusted for sex, ethnicity, marital status, wave of inclusion, education, occupational position, and number of morbidities at age 50. HR, hazard ratio. (TIF) [file pmed.1003147.s010.tif]
