# Supplementary material for: Healthy behaviors at age 50 years and frailty at older ages in a 20-year follow-up of the UK Whitehall II cohort: A longitudinal study
Source: PLoS Med. 2020 Jul 6;17(7):e1003147. doi: 10.1371/journal.pmed.1003147 (PMC7337284; doi:10.1371/journal.pmed.1003147)
Supplement: S1 Text — (DOCX) [file pmed.1003147.s011.docx]

**S1 Text. Prospective analysis plan**

The analysis undertaken in this manuscript was described in a proposal for an internship project submitted to the MSc of Research in Public Health, University Paris-Saclay in November 2018.

Please see below a translation of the MSc internship project initially written in French.

**Title:** Association between health behaviors at age 50 and risk of frailty during aging

**Background:**

Frailty is an age-related syndrome characterized by a high vulnerability to stressors and declines in functioning of various physiological systems, and is associated with increased risk of mortality, disability, falls and hospitalization (Fried et al. 2001). With the ageing of the population, it is important to maintain people in good health while avoiding deterioration of autonomy in activities of daily living in order to reduce the risk of hospitalisation or institutionalisation (Hillcoat-Nalletamby 2014, Mulasso et al. 2016, Op Het Veld et al. 2017). Health behaviors (smoking, alcohol consumption, diet, physical activity) are proposed as a key to healthy ageing (Vaes et al. 2017, Kehler 2018, Kojima et al. 2018, Singh-Manoux et al. 2018), and are also part of health promotion campaigns. However, their association with the risk of frailty has been little studied and mainly in elderly population (Cruz et al. 2017, Fhon et al. 2018). Furthermore, health behaviors change during aging, in part due to health conditions that may also influence the onset of frailty. It is therefore important to examine risk factors for frailty at an age when they are unlikely to be influenced by changes or risk factors that develop prior to the onset of frailty (e.g., multimorbidity) in order to avoid the risk of reverse causation.

**Objectives:**

The objective of this study is to examine the association of health behaviors, separately and in combination, measured at age 50 and the occurrence of frailty during aging using longitudinal data from the Whitehall II cohort.

**Data Availability:**

The study will be conducted on the Whitehall II database. This is a prospective longitudinal cohort of 10,308 women and men. The population consists of employees of the London offices of the British Civil Service and was recruited in 1985. Data were assessed based on clinical examinations and questionnaires. During the course of the study, 11 data collection waves were carried out between 1985 and 2015.

Health behaviors, assessed by questionnaire, will include: diet, alcohol consumption, physical activity and smoking habits.

Frailty will be based on the Fried's Frailty Scale (score from 0 to 5), which consists of self-reported measures of feeling tired and lack of physical activity, and clinical measures of involuntary weight loss (greater than 5kg per year), low muscle strength (measured from grip strength), and bradykinesia (slow walking speed) (Fried et al. 2001). Frailty will be defined as having at least 3 points on the Fried scale.

Numerous adjustment variables will be taken into account in the analyses such as socio-demographic, economic, and health measures.

**Methods:**

In order to assess the association of health behaviors, separately and in combination, with frailty onset during aging, "Illness-death" models will be used to account for interval censoring and competitive mortality risk. The outcome of interest will be frailty as a dichotomous variable, and the time until frailty is diagnosed.

**References**

Cruz DTD, Vieira MT, Bastos RR and Leite ICG (2017). "Factors associated with frailty in a community-dwelling population of older adults." Rev Saude Publica **51**: 106.

Fhon JRS, Rodrigues RAP, Santos JLF, Diniz MA, Santos EBD, Almeida VC and Giacomini SBL(2018). "Factors associated with frailty in older adults: a longitudinal study." Rev Saude Publica **52**: 74.

Fried L P, Tangen CM, Walston J, Newman AB, Hirsch C, Gottdiener J, Seeman T, Tracy R, Kop WJ, Burke G and McBurnie MA (2001). "Frailty in older adults: evidence for a phenotype." J Gerontol A Biol Sci Med Sci **56**(3): M146-156.

Hillcoat-Nalletamby S (2014). "The meaning of "independence" for older people in different residential settings." J Gerontol B Psychol Sci Soc Sci **69**(3): 419-430.

Kehler DS (2018). "The impact of sedentary and physical activity behaviour on frailty in middle-aged and older adults." Appl Physiol Nutr Metab **43**(6): 638.

Kojima G, Iliffe S, Jivraj S, Liljas A and Walters K (2018). "Does current smoking predict future frailty? The English longitudinal study of ageing." Age Ageing **47**(1): 126-131.

Mulasso A, Roppolo M, Giannotta F and Rabaglietti E (2016). "Associations of frailty and psychosocial factors with autonomy in daily activities: a cross-sectional study in Italian community-dwelling older adults." Clin Interv Aging **11**: 37-45.

Op Het Veld LPM, Ament BHL, van Rossum E, Kempen G, de Vet HCW, Hajema K and Beurskens A (2017). "Can resources moderate the impact of levels of frailty on adverse outcomes among (pre-) frail older people? A longitudinal study." BMC Geriatr **17**(1): 185.

Singh-Manoux A, Fayosse A, Sabia S, Tabak A, Shipley M, Dugravot A and Kivimäki M (2018). "Clinical, socioeconomic, and behavioural factors at age 50 years and risk of cardiometabolic multimorbidity and mortality: A cohort study." PLoS Med **15**(5): e1002571.

Vaes B, Depoortere D, van Pottelbergh G, Mathei C, Neto J and Degryse J (2017). "Association between traditional cardiovascular risk factors and mortality in the oldest old: untangling the role of frailty." BMC Geriatr **17**(1): 234.
