# Supplementary material for: Healthy behaviors at age 50 years and frailty at older ages in a 20-year follow-up of the UK Whitehall II cohort: A longitudinal study
Source: PLoS Med. 2020 Jul 6;17(7):e1003147. doi: 10.1371/journal.pmed.1003147 (PMC7337284; doi:10.1371/journal.pmed.1003147)
Supplement: Attachment — Submitted filename: response_letter_v2.docx [file pmed.1003147.s013.docx]

Editors

PLOS Medicine

25^th^ February, 2020

Dear editors,

**RE: Manuscript ID PMEDICINE-D-19-04222-R entitled “Healthy behaviors at age 50 years and frailty at older ages in a 20-year follow-up of the Whitehall II cohort: a longitudinal study"**

Thank you for giving us the opportunity to revise our paper. Please see below our detailed response to comments from the editorial board and the three reviewers. We hope that the revised version of our paper has addressed the concerns raised and is now suitable for publication.

We would like to thank you for considering our paper and look forward to hearing from you.

Yours sincerely,

Séverine Sabia, on behalf of all authors.

Université de Paris, Inserm U1153,

Epidemiology of Ageing and Neurodegenerative diseases,

10 avenue de Verdun, France

Telephone: +33 (0)1 57 27 90 46, Email: [severine.sabia@inserm.fr](mailto:severine.sabia@inserm.fr)

**EDITORS**

**1.** Please adapt the data statement to state "... because of constraints dictated by the study's ethics approval ..." or similar.

**OUR RESPONSE:** Thank you. We have now modified the sentence as suggested (page 28, line 487).

**2.** Please adapt the title to include a study descriptor and accord with journal style. We suggest "Healthy behaviors at age 50 years and frailty at older ages in a 20-year follow-up of the Whitehall II cohort: a longitudinal study".

**OUR RESPONSE:** Thank you for the suggestion. We have changed the title accordingly (page 1, lines 1-2).

**3.** Please add summary demographic information on participants to the abstract.

**OUR RESPONSE**: We have now added summary demographic information on participants as follows:

*“In this prospective cohort study of 6357 (29.2% women; 91.7% Caucasian) participants from the British Whitehall II cohort, healthy behaviors – non-smoking, moderate alcohol consumption, ≥2.5 hours per week of moderate-to-vigorous physical activity, and consumption of fruits or vegetables at least twice a day – were measured at age 50, and change in behaviors between 1985 (mean age=44.4) and 1997 (mean age=54.8).”* (page 2, lines 28-32)

**4.** In the abstract and elsewhere, please add p values alongside 95% CI where available.

**OUR RESPONSE:** As requested, p values are now provided alongside 95% CI in the full manuscript**.**

**5.** Please add a new final sentence to the "methods and findings" subsection of your abstract to summarize the study's main limitations.

**OUR RESPONSE:** We have added details of the main limitations of the study as follows:

*“The primary limitation of this study is potential selection bias during the follow-up due to missing data on frailty components.”* (page 3, lines 48-49)

**6.** At line 45, please begin the sentence with "Our findings suggest that ..." or similar.

**OUR RESPONSE:** Thank you. As suggested, the conclusion now reads as follows:

*“Our findings suggest that healthy behaviors at age 50, as well as improvements in behaviors over midlife, are associated with a lower risk of frailty later in life. Their benefit accumulates so that risk of frailty decreases with greater number of healthy behaviors. These results suggest that healthy behaviors in midlife are a good target for frailty prevention.”* (page 3, lines 50-53)

**7.** After the abstract, we will need to ask you to add a new and accessible "author summary" section in non-identical prose. You may find it helpful to consult one or two recent research papers published in PLOS Medicine to get a sense of the preferred style.

**OUR RESPONSE:** We have added the author summary after the abstract, see pages 4-5.

**8.** Early in the methods section of your main text, please state whether the study had a protocol or prespecified analysis plan and if so attach the document(s) as a supplementary file (referred to in the text). Please highlight analyses that were not prespecified.

**OUR RESPONSE:** Analyses related to this study were proposed for an MSc internship project for which we now provide a translation from the French version that was advertised in November 2018. We refer to this document in the second sentence of the Methods section as follows (page 7, lines 117-119):

*“The study objectives and analysis plan were developed prior to data manipulation for an MSc internship (S1 Text). The analyses referred to as post-hoc analyses were in response to suggestions from reviewers.”*

**9.** In the discussion of study limitations at line 281, please add some additional detail. For example, in noting the limitations of observational evidence, you could mention the possible existence of unmeasured confounders.

**OUR RESPONSE:** Thank you, the sentence now reads (page 23, line 399):

*“Four, the use of observational data cannot rule out the role of unmeasured confounders.”*

**10.** Throughout the text, please style reference call-outs as follows: "... involved in frailty development [49,50]." (i.e., preceding punctuation).

**OUR RESPONSE:** We followed the editors’ recommendations and modified the reference call-outs in the manuscript as suggested.

**11.** In the reference list, please ensure that journal names are abbreviated correctly and consistently (e.g., "Lancet" for references 2, 4 and 8).

**OUR RESPONSE:** We have now revised the reference list to match with the journal guidelines and harmonized the abbreviations of journal names.

**12.** Please additional access details to reference 10.

**OUR RESPONSE:** This reference refers to a report from the European commission for which we have added the link to the website (<https://ec.europa.eu/research/innovation-union/pdf/active-healthy-ageing/gp_a3.pdf>), please see reference 10.

**13.** Please add a completed checklist for the most appropriate reporting guideline, which we suspect will be STROBE, as a supplementary document (referred to in the methods section). In the checklist, individual items should be referred to by section (e.g., "Methods") and paragraph number rather than by page or line numbers, as the latter generally change in the event of publication.

**OUR RESPONSE:** We have revised the STROBE checklist to match the paragraph numbers and sections from the manuscript rather than page or line numbers.

**REVIEWER 1**

**1.** "Association between healthy behaviors at age 50 and frailty at older ages: 20-year follow-up of the Whitehall II cohort study" analyzes the well-known Whitehall II cohort of British civil servants, in particular as to how "healthy behaviors" at age 50 affect "frailty" approximately 20 years later (with a range of plus or minus five years from the starting age, and also with a range for the follow-up depending on which of the four later clinical evaluations (2002, 2007, 2012, 2015) was most appropriate. The general findings were unsurprising, in that exhibiting more examples of healthy behaviours at age 50 correlated with reduced frailty later on. The hazard ratio for each additional healthy behaviour was estimated to be 0.69, and for all vs. no healthy behaviours as 0.28.

**OUR RESPONSE:** We thank the reviewer for a nice summary of our paper.

**2.** The authors used an appropriate interval-censored illness-death model for the main statistical analysis. Furthermore, the expected additional demographic and socioeconomic covariates were considered. On the whole, the analysis and conclusions seem broadly valid. However, there remain a few points that might be considered.

**OUR RESPONSE:** We thank the reviewer for this positive feedback. We have revised the paper to include the reviewer’s suggestions, please see our response below to specific comments.

**3.** On the variability of the cohort participants in terms of initial age and follow-up age, it might be helpful to include more detailed data, such as a table with rows as initial age and columns as years to follow-up, with each cell containing the number of participants with that initial age/years to follow-up combination. Further on the data, while the demographic and healthy behaviour characteristics are provided in Table 1, there is no such information for frailty characteristics. This could be added.

**OUR RESPONSE:** We thank the reviewer for these suggestions. We have now added a table in supplementary materials with details of age and wave at inclusion in the analysis as well as follow-up information (S1 Table).

This is described in the results section as follows (page 12, lines 240-245):

*“Thus, the final analytic cohort included 6357 participants, of whom 445 (7%) became frail at a mean age of 72.1 (standard deviation (SD), 6.9) years and 701 (11%) died over a mean follow-up of 20.4 (SD, 5.9; range, 3.8 to 31) years. Further description of the composition of the analytic sample, corresponding to when participants were aged 50 (mean age=50.4 (SD=2.1) years), and corresponding mean follow-up are provided in S1 Table.”*

As suggested, we have also added information on frailty components in Table 1. This is described in the results section as follows (page 13, lines 254-255):

***“****At the end of follow-up, each frailty component, apart weight loss, was present in over 60% of the frail group (Table 1).”*

**4.** On "Moderate alcohol consumption" being defined as a healthy behavior (line 95), the referenced source [32] states that "no robust association with alcohol consumption was found". In any case, it is unclear why "None" is not also defined as healthy behavior. As such, more involved analysis might be performed on alcohol consumption (e.g. comparing "None" only, and "None+Moderate" as criteria)

**OUR RESPONSE:** We would like to apologize for the reference that was erroneous. We have now changed the reference in the text and refer to a recent paper reporting a non-linear association between alcohol consumption and frailty onset, with moderate drinkers having the lowest risk of frailty incidence (reference 23 in the manuscript, Kojima et al, Bioscience trends, 2017). This is also suggested by our results in Table 2 showing lower risk of frailty among moderate drinkers in adjusted models, confirming the non-linear association we initially found in exploratory analysis where we looked at the shape of the association between alcohol consumption and frailty onset using spline regression. We now present results from this exploratory analysis in S1 Figure.

As suggested, we have also undertaken a post-hoc analysis to examine alternative categorizations for healthy alcohol consumption defined as “no alcohol consumption” and “no or moderate alcohol consumption”. Results are presented in S6 Table and reported in the Results section as follows (page 22, lines 352-356):

*“Associations between alternative definitions of healthy alcohol consumption and frailty onset are presented in S6 Table. In fully adjusted models, as compared to “moderate or high alcohol consumption”, “no alcohol consumption” was associated with higher risk of frailty (HR 1.36, 95%CI 1.08 to 1.70; p=0.008) and there was no statistically significant difference in risk of frailty between “no or moderate alcohol consumption” and “high alcohol consumption” (HR 0.83, 95%CI 0.66 to 1.06; p=0.14).”*

*Reference cited*

- *Kojima G, Iliffe S, Liljas A, Walters K. Non-linear association between alcohol and incident frailty among community-dwelling older people: A dose-response meta-analysis. Bioscience trends. 2017;11(5):600-2.*

**5.** A general comment on the categorizations used for frailty and healthy behavior measures: while various justifications are cited (e.g. most prominently Fried's frailty phenotype for frailty), the availability of raw values for at least some of these measures suggests that more nuanced analyses might be possible for those measures at least. For example, the "slow walking speed" measure is suddenly discontinuous at a height of 173/159cm for males and females respectively. It could be interesting to examine effects with greater precision (e.g. for "Moderate alcohol consumption", the range of 1 to 14 units is also rather large and could conceivably be split further). Of course, it remains up to the authors as to whether these might constitute reasonable secondary analyses.

**OUR RESPONSE:** Papers from the Whitehall II study (Brunner et al, Lancet Public Health, 2018; Dugravot et al, Lancet Public Health, 2020) and other studies on frailty (for example in the UK Biobank cohort, Hanlon et al, Lancet Public Health, 2018) use the original cut-offs for Fried’s frailty criteria as this allows comparison of findings across studies. The Whitehall study is an occupational cohort and the health profile of participants is better than the general population although the association of risk factors with health outcomes is broadly similar to that in the general population (Batty et al, Epidemiology, 2014). Thus, we feel that using study specific thresholds is not meaningful and would add noise to the research literature on frailty.

Regarding more detailed categorisation of health behaviors, we examined the shape of the continuous health behavior variables, alcohol consumption and physical activity, with frailty onset using cubic spline regressions in exploratory analysis previous to the original submission. We now present results from these analyses in Figures S1 and S2 respectively. Results were in accordance with categorization of these health behaviors in previous studies that examined associations with various health outcomes (for examples, Sabia et al, J Am Geriatr Soc, 2014; Khaw KT et al, PLoS Med, 2008). Thus we decided to use these categorisations to allow comparability with other studies as now described into the Methods section, paragraph on health behaviors at age 50 (page 8, lines 140-146).

*References cited*

- *Brunner E, Shipley MJ, Ahmadi-Abhari S, Valencia Hernandez C, Abell JG, Singh-Manoux A, Kawachi I, Kivimäki M. Midlife contributors to socioeconomic differences in frailty during later life: a prospective cohort study. Lancet Public Health. 2018;3(7),e313-e322.*
- *Hanlon P, Nicholl B, Dinesh Jani B, Lee D, Mc Queenie R, Mair FS. Frailty and pre-frailty in middle-aged and older adults and its association with multimorbidity and mortality: a prospective analysis of 493737 UK Biobank participants. Lancet Public Health, 2018;3(7):e323-e332.*
- *Batty GD, Shipley M, Tabák A, Singh-Manoux A, Brunner E, Britton A, Kivimäki M. Generalizability of occupational cohort study findings. Epidemiology. 2014 Nov;25(6):932-3.*
- *Sabia S, Elbaz A, Rouveau N, Brunner EJ, Kivimaki M, Singh‐Manoux A. Cumulative associations between midlife health behaviors and physical functioning in early old age: a 17‐year prospective cohort study. J Am Geriatr Soc. 2014;62(10):1860-8.*
- *Khaw K-T, Wareham N, Bingham S, Welch A, Luben R, Day N. Combined impact of health behaviours and mortality in men and women: the EPIC-Norfolk prospective population study. PLoS Med. 2008;5(1):e12.*

**REVIEWER 2**

**1.** This original article evaluates the association between health behaviors at age 50 in Whitehall II cohort study participants and the incidence of the frailty syndrome, using Fried's phenotype, at 4 follow-up visits, in 2002, 2007, 2012 and 2015. The findings show that each healthy behavior was independently associated with lower risk of incident frailty, and that the impact of aggregate health behaviors was cumulative in further lowering the risk for frailty.

**OUR RESPONSE:** Thank you for this nice summary of the study.

**2.** Methods: p. 4: Baseline recruitment occurred in 1985-88, when those recruited were 35-55; it would be useful to know the range of length of follow-up after age 50 for those who were included in this analysis. Mean follow up length is mentioned on p. 16 as being 20 years.

**OUR RESPONSE:** We thank the reviewer for this suggestion. As also suggested by Reviewer 1 (comment 3), we have added a supplementary table to describe the length of follow-up as a function of wave at inclusion, corresponding to the wave when participants were aged 50. This is described in the Results section as follows (page 12, lines 240-245):

*“Thus, the final analytic cohort included 6357 participants, of whom 445 (7%) became frail at a mean age of 72.1 (standard deviation (SD), 6.9) years and 701 (11%) died over a mean follow-up of 20.4 (SD, 5.9; range, 3.8 to 31) years. Further description of the composition of the analytic sample, corresponding to when participants were aged 50 (mean age=50.4 (SD=2.1) years), and corresponding mean follow-up are provided in S1 Table.”*

**3.** Results: Given that p. 9 indicates confounding by higher SES and fewer comorbidities among those with greater number of healthy behaviors at age 50, it would be useful to understand if those with lower socio economic background who also had positive health behaviors had comparable outcomes to those of higher socioeconomic background. Further, is it possible to offer an analysis of length of exposure to adverse behaviors prior to age 50, by confounders?

**OUR RESPONSE:** In order to assess whether healthy behaviors show similar association with frailty onset in the low compared to the high socioeconomic group, we performed interaction tests of healthy behaviors with education and occupational position. None of the interactions were statistically significant suggesting that the association between healthy behaviors and frailty onset does not differ by socioeconomic background. This finding is now reported in the results section as follows (page 15, lines 263-264):

*“As there was no evidence of an interaction of the number of healthy behaviors with sex (p=0.22), education (p=0.47) and occupational position (p=0.14), analyses were undertaken in the total population.”*

Regarding length of exposure to adverse behaviors, as also suggested by reviewer 3 (comment 3), we have now added analysis on change in health behaviors during midlife, as assessed by change in health behaviors between 1985 and 1997, and subsequent frailty onset to the revised manuscript. These results are presented in Table 4 and discussed in the Results section as follows (page 20, lines 336-345):

*“Change in the number of healthy behaviors was calculated among 6435 participants with data on health behaviors available in 1985 and 1997 (N=5380) [or 1991 (N=905) or 2002 (N=150) when data in 1997 were missing]. This corresponds to change over a mean of 10.4 (SD=2.4) years, with mean age being 44.4 (SD=5.9) years at the first measure and 54.8 (SD=6.4) years at the second measure. The mean number of healthy behaviors increased by 0.4 (SD=1.0) during this 10-year period. In a fully adjusted illness-death model, with no or 1 healthy behavior in both 1985 and 1997 as the reference group, HR for 2 healthy behaviors at both measures was 0.57 (0.41 to 0.81; p=0.001), and 0.32 (0.23 to 0.46; p<0.001; Table 4) for 3-4 healthy behaviors at both measures. Among those with no or 1 healthy behavior in 1985, change to 2 (HR 0.64 95%CI 0.44 to 0.94; p=0.02) or 3-4 (0.57 95%CI 0.38 to 0.87; p=0.009) healthy behaviors in 1997 was associated with lower risk of frailty compared to 0 or 1 healthy behavior in 1997 (Table 4).”*

**4.** p. 11: could the comparable incidence of frailty for moderate and high alcohol drinkers be associated with loss to mortality among the high drinkers?

**OUR RESPONSE:** This is an interesting point. Incidence rates reported in the text are unadjusted. Hazard ratio reported in the tables were adjusted for covariates and accounted for death as a competing risk. We have now clarified this in the Methods section as follows (page 10, lines 188-200):

*“In descriptive analysis, unadjusted frailty incidence rates per 1000 person-years were calculated for each category of health behaviors. Participants contributed to person-years from the wave they were aged 50 to the first wave they were diagnosed as frail or their last clinical examination whichever was first.*

*Frailty was assessed at clinical evaluations in 2002, 2007, 2012, and 2015 but the exact date of frailty onset was unknown (interval-censored data). In addition, some participants may have developed frailty and died between two data collection waves without being identified as frail, making death a competing event. We therefore used an interval-censored illness-death model with a Weibull distribution to assess hazard ratio (HR) of frailty with respect to health behaviors categories. This analysis accounts for both the interval-censored nature of the data and competing risk of death [36].”*

As reported in the Results section (page 15, line 269-275), there was no statistically significant difference in unadjusted incidence rates between moderate and high consumption of alcohol, however when accounting for demographic factors and competing risk of death, moderate alcohol consumption was associated with a lower risk of frailty compared to high alcohol consumption (HR=0.76, 95%CI 0.59 to 0.97; p=0.03; Table 2).

**5.** p. 11: associations attenuated after adjustment for socioeconomic factors, comorbidities. Is the association of occupational position with frailty attenuated by healthy behaviors, and if so which?

**OUR RESPONSE:** The focus of our paper is on the association between health behaviors and frailty onset. Occupational position is considered in the analysis as a confounding factor (Model 2, 3, and 4 are adjusted for occupational position). As suggested by this reviewer (comment 3), we also examined whether occupational position modified the association between health behaviors and frailty and found it not to be the case (Results section, page 15, lines 263-264). However, we feel it may be beyond the scope of the paper to assess whether health behaviors explain socioeconomic disparities in frailty onset. In fact, this has already been reported in a previous paper from the Whitehall II study (Brunner et al, Lancet Public health, 2018) where the authors found each health behavior to contribute to the association between occupational position and frailty with the largest mediation observed for physical activity (the association between occupational position and frailty reduced by 13.4% after adjustment for physical activity, see Table 4 from this paper copied below).

*Reference cited*

- *Brunner E, Shipley MJ, Ahmadi-Abhari S, Valencia Hernandez C, Abell JG, Singh-Manoux A, Kawachi I, Kivimäki M. Midlife contributors to socioeconomic differences in frailty during later life: a prospective cohort study. Lancet Public Health. 2018;3(7),e313-e322.*

**6.** Discussion: It would be useful to hear analysis of the finding on p. 11 that there was no evidence of interaction between sex and number of healthy behaviors in association with frailty.

**OUR RESPONSE:** We thank the reviewer for this suggestion. We have now added the following text to the Discussion section (page 25, lines 441-444):

*“Frailty is more prevalent among women and participants from the lower socioeconomic group; in our analyses the number of healthy behaviors at age 50 and the change in this number over midlife were similarly associated with risk of incident frailty in both sexes and in different socioeconomic groups.”*

**7.** p. 18. Lines 309-312. The aggregate health outcomes affected by these health behaviors warrants further discussion. Since those with positive health behaviors had fewer comorbidities at age 50, please discuss the findings re: independence of association of health behaviors and frailty from presence of chronic diseases, comorbidity.

**OUR RESPONSE:** In the present paper, we adjusted the analysis for prevalence of morbidities assessed at the time of health behaviors measurements at age 50 (Tables 2, 3 and 4, Figure 2) in order to control for confounding by chronic conditions on the association between health behaviors and incident frailty. However, as health behaviors are associated with incidence of several chronic conditions that in turn are risk factors for frailty, chronic conditions can also mediate the association between health behaviors and incidence frailty. This is now highlighted in the Discussion section, page 25 lines 432-436, as follows:

***“****Furthermore, unhealthy behaviors are associated with higher incidence of multiple chronic diseases [13] known to increase the risk of frailty [28], making chronic conditions potential intermediaries of the association between health behaviors and frailty onset. Indeed, in the present study, 10.6% of participants who developed frailty during the follow-up had at least two morbidities already at age 50 and this proportion reached 47.9% at the time of frailty onset.”*

**REVIEWER 3**

**1.** Thank you for the opportunity to review the manuscript by Gil-Salcedo who investigated the association of health behaviors with developing physical frailty in adults who were 50 years old at baseline. Midlife represents a critical time in which healthy lifestyle behaviors could play a major role in helping to prevent the health problems of old age (e.g., dementia PMID: 28735855) and understanding health behaviors may be pivotal in frailty prevention (PMID: 26805753). In this study, participants from the Whitehall II cohort study (n= 6357) had their smoking status, alcohol consumption, physical activity, and fruit/vegetable consumption measured at their first study visit (at age 50). Frailty was assessed at four time points, over a period of 20 years. All health behaviors were independently associated with developing frailty (445/6357 developed frailty) even when all health behaviors were included in their model which considered death as a competing risk. Non-smokers had the lowest risk of developing frailty, followed by moderate alcohol consumption, physical activity, and then fruit/vegetable consumption. A greater number of health behaviors resulted in a dose-associated lower risk of frailty.

**OUR RESPONSE:** We thank the reviewer for this nice summary of our paper.

Major comments:

**2.** Have the authors considered examining sedentary time in their models? Sedentary behaviors (low energy expenditure activities while seated/lying/reclining during awake hours) are associated with frailty (PMID: 30355522) and may provide insights into their data. It might be possible that even though someone is physically active, they could still report high levels of sitting time.

**OUR RESPONSE:** Thank you for raising this interesting point. Information on sedentary time was assessed only once over the follow-up period in 1997 when participants were aged between 45 and 69 years. These data did not allow us to extract a measure of sedentary behavior at age 50 for the entire study sample. Thus, we undertook post-hoc analysis to examine the association between sedentary time in 1997 and incident frailty. This is reported as follows in the manuscript:

Methods section (page 11, lines 226-233)

*“Three, we undertook exploratory analysis on sedentary time as an additional health behavior. Sedentary time was assessed only in 1997 and could not be extracted at age 50, thus we examined the association between sedentary time in 1997 and incident frailty. Sedentary time was measured as time spent sitting, calculated based on self-reported number of hours per week spent “sitting at work, driving, commuting or other” and “sitting at home e.g. watching TV, sewing, at desk”, and was categorized into tertiles given the lack of definition of unhealthy sedentary time. A score of healthy behavior was calculated with all 5 behaviors, with healthy sedentary time defined as not being in the highest tertile of time spend sitting. “*

Results section (page 22, lines 357-364)

*“Post-hoc analysis on the association between sedentary time in 1997 and incident frailty based on 5296 participants (N incident frailty = 351) are presented in S7 Table. In analysis adjusted for demographic factors, sitting time in the first (<2.8 hours/day; HR 0.74, 95%CI 0.57 to 0.96; p=0.02) and the second (2.8 to 4.4 hours/day; HR 0.77, 95%CI 0.59 to 1.00; p=0.05) tertiles were associated with lower risk of frailty compared to sitting time in the top tertile (>4.4 hours/day). In fully adjusted analysis (including other healthy behaviors in 1997), the HR of frailty associated with healthy sitting time (<4.4 hours/day) was 0.79 (95% CI 0.63 to 0.99; p=0.04). When healthy sitting time was included in the score of healthy behaviors (range 0 to 5), HR per 1 additional healthy behavior was 0.72 (95%CI 0.65 to 0.81; p<0.001).”*

Discussion (pages 25-26, lines 451-455)

*“Research on health behaviors would benefit from more precise and objective measures as much of the research, including ours, remains based on self-reported behaviors. For example, accelerometers are increasingly being used for objective assessment of physical activity and sedentary behavior [52]. A further area of research is better assessment of sedentary behavior, our post-hoc analysis suggests an independent role of sedentary behavior on frailty onset.”*

**3.** A novelty of this study is the examination of the impact of multiple health behaviors on developing frailty. However, it is reasonable to assume that health behaviors changed over the follow up period in the present study, and such changes are likely to impact whether an individual develops frailty or not. Based on the author's description of the Whitehall II study, it is feasible to assess changes in health behaviors. It is strongly recommended that changes in health behaviors be assessed in relation to developing frailty, as these results would be quite novel.

**OUR RESPONSE:** We thank the reviewer for this excellent suggestion. We have now added post-hoc analysis to assess the association between change in the number of healthy behaviors over midlife and incident frailty to the revised manuscript.

This analysis is described in the Methods section (page 11, lines 219-225) as follows:

*“One, data on health behaviors assessed in 1985 and 1997, at mean ages 44.4 and 54.8 years, were used to examine the association of change in health behaviors and subsequent frailty. The number of healthy behaviors was categorized into 3 groups (0-1, 2, and 3-4) at each time point and change in these categories was examined in the analysis. Participants were followed for incident frailty from 1997 to the end of follow-up. Covariates in the analysis were assessed in 1997. For participants with missing data in 1997, data on health behaviors and covariates from 1991 or 2002 were used.”*

Results are presented on pages 20, lines 336-345:

*“Change in the number of healthy behaviors was calculated among 6435 participants with data on health behaviors available in 1985 and 1997 (N=5380) [or 1991 (N=905) or 2002 (N=150) when data in 1997 were missing]. This corresponds to change over a mean of 10.4 (SD=2.4) years, with mean age being 44.4 (SD=5.9) years at the first measure and 54.8 (SD=6.4) years at the second measure. The mean number of healthy behaviors increased by 0.4 (SD=1.0) during this 10-year period. In a fully adjusted illness-death model, with no or 1 healthy behavior in both 1985 and 1997 as the reference group, HR for 2 healthy behaviors at both measures was 0.57 (0.41 to 0.81; p=0.001), and 0.32 (0.23 to 0.46; p<0.001; Table 4) for 3-4 healthy behaviors at both measures. Among those with no or 1 healthy behavior in 1985, change to 2 (HR 0.64 95%CI 0.44 to 0.94; p=0.02) or 3-4 (0.57 95%CI 0.38 to 0.87; p=0.009) healthy behaviors in 1997 was associated with lower risk of frailty compared to 0 or 1 healthy behavior in 1997 (Table 4).”*

Findings are also discussed in the Discussion and Conclusion section as follows:

*“The present study using repeated measures of health behaviors also adds to the current literature by suggesting that favorable changes in lifestyle during midlife might reduce the risk of frailty at older ages.”* (page 24, lines 424-426)

*“Our analysis based on an observational cohort study showed that a greater number of healthy behaviors at age 50 as well as improvement in health behaviors over midlife were associated with lower risk of frailty over a 20-year follow-up period. These findings highlight the importance of healthy behavior in midlife for prevention of frailty at older ages.”* (page 26, lines 457-460)

**4.** The authors adjust for several chronic conditions at age 50 in their statistical models, some of which are more severe than others. Can the authors determine which baseline conditions were more likely to result in developing frailty?

**OUR RESPONSE:** In the present analysis, we chose to adjust for the number of morbidities at age 50 to account for the burden of disease rather than consider each condition separately as the prevalence of each condition at age 50 was quite low. We have now added S2 Table to present the prevalence of each chronic condition at age 50 by frailty at the end of follow-up. Participants who developed frailty were more likely to be obese and have a history of depression at age 50.

Minor comments:

**5.** It appears that disability was not considered in their models. How might they affect the findings here? Were participants free of disability at baseline?

**OUR RESPONSE:** Participants from the Whitehall II study were all in full-time employment at study inception. We assessed health behaviors at age 50 years, making the assumption that they were not likely to be disabled. Information on disability was recorded only from 2006 onwards and thus we were not able to test this assumption. We have added this limitation to the Discussion section as follows (page 23, lines 399-401):

*“Finally, disability status at age 50 was not available but as all participants were in full-time employment the likelihood of confounding bias by disability might be small.”*

**6.** Can the authors please comment on the multiple frailty tools which exist? Although the frailty phenotype is among the most widely used in research, how might the findings differ if another frailty tool, say the frailty index based on the accumulation of deficits model, were used?

**OUR RESPONSE:** There are a large number of tools to assess frailty as summarized in a paper by Aguayo et al (PLoS Med, 2018) which shows the association of 35 measures of frailty with subsequent mortality. All these measures predicted mortality with the Fried index showing among the strongest association. In addition, compared to other measures such as the accumulation of deficits model that comprise up to 70 components depending on the definition, the Fried’s frailty phenotype has the advantage of being parsimonious as it is based only on 5 components. We thus made the choice to use this measure. It allows replication across a wide range of studies and is also easily implementable in clinical setting. This is now highlighted in the Discussion section as follows (page 25, lines 450-454):

*“Among the multiple tools to assess frailty, we chose to use Fried’s frailty phenotype given its robust association with health outcomes, its use in several studies making our results amenable to replication, and the fact that its association with health outcomes such as mortality has been shown to be similar to that of more extensive measures such as the frailty index based on the accumulation of deficits model [51].”*

Reference cited

- Aguayo GA, Vaillant MT, Donneau AF, Schritz A, Stranges S, Malisoux L, Chioti A, Guillaume M, Muller M, Witte DR. Comparative analysis of the association between 35 frailty scores and cardiovascular events, cancer and total mortality in an elderly general population in England: An observational study. PLoS Med 2018;15(3):e1002543.

**7.** It is recommended that the authors should comment on the implications of their findings relation to public health and in the clinical context.

**OUR RESPONSE:** Thank you for the suggestion, we have added a section to the discussion on Clinical implications and future research and modified the Conclusion. Please see pages 25-26, lines 437-460.
