# Supplementary material for: Healthy behaviors at age 50 years and frailty at older ages in a 20-year follow-up of the UK Whitehall II cohort: A longitudinal study
Source: PLoS Med. 2020 Jul 6;17(7):e1003147. doi: 10.1371/journal.pmed.1003147 (PMC7337284; doi:10.1371/journal.pmed.1003147)
Supplement: Attachment — Submitted filename: response_letter_R2.docx [file pmed.1003147.s014.docx]

Editors

PLOS Medicine

7^th^ May, 2020

Dear editors,

**RE: Manuscript ID PMEDICINE-D-19-04222-R1 entitled “Healthy behaviors at age 50 years and frailty at older ages in a 20-year follow-up of the UK Whitehall II cohort: a longitudinal study"**

Thank you for considering accepting the present paper for publication. Please see below our response to comments from the editorial board, the reviewers, and the production team. We hope that the revised version of our paper is now suitable for publication.

We would like to thank you for considering our paper.

Yours sincerely,

Séverine Sabia, on behalf of all authors.

Université de Paris, Inserm U1153,

Epidemiology of Ageing and Neurodegenerative diseases,

10 avenue de Verdun, France

Telephone: +33 (0)1 57 27 90 46, Email: [severine.sabia@inserm.fr](mailto:severine.sabia@inserm.fr)

**EDITORS**

1- Please consider addding "UK" to the title: "Healthy behaviors at age 50 years and frailty at older ages in a 20-year follow-up of the UK Whitehall II cohort: a longitudinal study"

**OUR RESPONSE:** thank you, we followed your advice and changed the title as requested.

2- In the "Study population" section of the Methods, please specify whether consent is written.

**OUR RESPONSE:** we have added the information as follows:

*“Participant written, informed consent and research ethics approvals are renewed at each contact; the latest approval was by the NHS London - Harrow Research Ethics Committee, reference number 85/0938.”*

3- In the Discussion, please switch the order of the “Strengths and limitations” and “Comparison with previous studies” sections.

**OUR RESPONSE:** we have now switched the order of the two sections.

4- Regarding your response to reviewer 2, point 5, it may be relevant to mention this point and reference the Brunner study in the Discussion.

**OUR RESPONSE:** thank you for the suggestion, we have added the following text, in italic, to the Discussion section:

*“*Our findings highlight the potential key role of healthy behaviors in midlife for frailty prevention later in life with risk reduction ranging from 24% to 44% depending on the health behavior considered*.* *These associations were adjusted for socioeconomic factors, but it is worth noting that health behaviors also contribute to the association between socioeconomic advantage and frailty as shown in previous analysis from the Whitehall II cohort study.[37]”*

**REVIEWERS**

**Reviewer #1:** We thank the authors for carefully considering our previous comments, and performing appropriate post-hoc analyses. The remaining minor suggestions pertain mostly to the supplementary material:

1. There appears to be an issue with the image for S3 Figure (green background, blurred words)

**OUR RESPONSE:** In the submitted zipped folder from the Plos Medicine submission website, the file S3 Figure does not appear to be problematic. The issue might have appeared at the downloading from the reviewer side. We would be grateful if the editor could let us know whether the problem persists.

2. For Table 2/3 & S4 to S7 Tables, it might be helpful for the N frail/N total ratio to also be expressed as a percentage for easy comparison, if formatting permits.

**OUR RESPONSE:** We have now added the percentage to the tables as requested.

**Reviewer #3:** The reviewers have done a great job addressing reviewer comments. I accept the article in its revised form.

**OUR RESPONSE:** We thank the reviewer for this feedback.

**PRODUCTION**

FIGURES and GRAPHICS

1. Please remove embedded figures from your manuscript file and ensure the most up to date versions of your figures are uploaded to our submission system as separate TIF or EPS files. Please do not remove the figure legends and captions.

**OUR RESPONSE:** We have now removed the figures for the manuscript file, keep the title and legend.

2. Please ensure that all main figure files are cited in ascending numerical order in the main text of the article.

**OUR RESPONSE:** This is done.

3. Please ensure that all main figures are referenced in the manuscript as Fig 1, Fig 2, etc. (including capitalization), rather than Figure 1, Figure 2, etc. Please note, however, that the file names themselves must not include the space, i.e. Fig1.tif, Fig2.tif, etc.

**OUR RESPONSE:** This is now modified as requested.

4. Please ensure that all main figure legends are placed after the paragraph containing their first in-text citation.

**OUR RESPONSE:** This is done.

5. Figure 2 is low resolution and will appear blurry or pixelated if published. Text and images should appear crisp and clear. Please note that this problem will not be detected or corrected by the PACE tool, and this will not be fixed by simply increasing the resolution of your Figures in the image editing software. Please carefully review the PLoS Figure Guidelines and provide Figure files that were originally created at 300 pixels/inch (dpi) and at least 6.68cm wide, but less than 19.05cm and under 22.225cm tall.

1) Export a PDF from PowerPoint/Word/Excel (or other image source file). On a Mac, click on the 'PDF' button in the printer dialog box and select 'Save as PDF'. On a PC, send the file to print and select 'Adobe PDF' or 'PDFCreator' (it's freeware) from your list of available printers.

If you are using Adobe PDF, then go to Printer Properties, Default Settings and select Press Quality.

If you are using PDFCreator, Go to 'Printer Properties' and then click on 'Advanced' on the bottom right. Under 'Graphic', go to 'Print Quality' and select 600dpi.

2) PHOTOSHOP: When you open up the PDF, make sure that the 'Constrain Proportions' box is checked. Set the 'Resolution' to 300dpi (118.11 pixels/cm) and set the 'Bit Depth' to 8 bit. Use the crop tool (next to the eyedropper) to remove unnecessary white space. Go to - Image - Image Size. Make sure the 'Constrain Proportions' and 'Resample Image” boxes are checked.

To ensure that your Figures publish properly, please delete the alpha channels from each Figure file. In Photoshop, select it in the Channels Palette and click on the trash can icon. When you save the TIFFs make sure that the 'LZW' button is checked and 'Discard Layers and Save a Copy' button is checked.

3) GIMP: When you open up the PDF, set the resolution to 300. Make sure the chain link icon is joined together. Then change the size of your figure according to the pixel specifications mentioned in step 2) above. To ensure that your Figures publish properly, please delete the alpha channels from each Figure file. In GIMP, go to Layers - Transparency - Remove Alpha Channel. When you are done, 'SAVE AS' (or Export if GIMP 2.8). Select File Type - 1st TIFF option. Make sure to use LZW compression.

4) Print out your figures on a desktop printer. Text and graphics should appear clear and crisp with no pixelated or blurred edges.

5) If the resulting figures are still blurry, then your original source images are too low of a resolution. Please increase the resolution of your original source images, and repeat this process.

**OUR RESPONSE:** We have now uploaded a new version of Figure 2.

6. Figures 1 and 2 contain font size that is below our recommended range of font sizes. Please note that this problem will not be detected or corrected by the PACE tool. We ask that all type be between 8-12 point (2.8 - 4.2 mm). It is still possible to read 5-6 point type but should only be used sparingly in your figure. For example, it is acceptable for numbers along an axis of a graph or for subscripts and superscripts. 4 point, or smaller, text is not allowed. We recommend that you print figures out to get a better idea of the text legibility.

**OUR RESPONSE:** We have now uploaded a new version of the figures.

TABLES:

1. Table 4 uses returns to control text wrapping. This is indicated by a [¶ or ↵] symbol when "Show Paragraph Marks" is turned on in Word. Please either split those cells into separate cells to achieve alignment or remove the returns.

**OUR RESPONSE:** Now corrected.

METADATA:

1. Please ensure that the CRediT author contributions listed for each co-author are completed in full. To qualify for authorship, each author must meet at least one of the seven core contributions (conceptualization; data curation; formal analysis; investigation; methodology; software; validation), as well as at least one of the writing contributions (original draft preparation; review and editing). Authors may also be noted against any of the remaining contributions, however these alone will not qualify a contributor for authorship.

Please ensure that the listed contributions for each author qualify them for authorship based on the above criteria. Please ensure that the full contributions of each author are acknowledged in the "Add/Edit/Remove Authors" section of our submission form. If they do not meet criteria for authorship please let us know as soon as possible by emailing [plosmedicine@plos.org](mailto:plosmedicine@plos.org).

**OUR RESPONSE:** Thank you, the author contributions is now completed in full on the online submission form.

2. Please provide a detailed Financial Disclosure statement on the submission form in full sentences, and include URLs, the initials of the receiving author, and specific grant numbers wherever possible.

**OUR RESPONSE:** The Financial Disclosure statement has been provided as requested on the submission form.

3. Please ensure that the funders and grant numbers match between the Financial Disclosure field and the Funding Information tab in your submission form. Note that the funders must be provided in the same order in both places as well.

**OUR RESPONSE:** We have now ensured that information in both places match.

4. In accordance with PLOS' data policy, please ensure that your Data Availability Statement in the submission form clearly identifies how readers can access your data. Data cannot be available on request, or only accessible by contacting one of the authors. Note that your Data Availability Statement will be typeset as it is written, so please ensure it is in complete sentences and appears as you would like it to in the published version.

For details on our data policy, please see <http://journals.plos.org/plosmedicine/s/data-availability>

**OUR RESPONSE:** The statement reads as we would like it to appear.

5. In the online submission form, you indicated that "data cannot be shared publicly because of constraints dictated by the study's ethics approval. The Whitehall II data are available for sharing within the scientific community". All PLOS journals now require all data underlying the findings described in their manuscript to be freely available to other researchers, either 1. In a public repository, 2. Within the manuscript itself, or 3. Uploaded as supplementary information.

This policy applies to all data except where public deposition would breach compliance with the protocol approved by your research ethics board. If your data cannot be made publicly available for ethical or legal reasons (e.g., public availability would compromise patient privacy), please explain this by return email and staff will assist you with completing your Data Availability Statement.

At this time, we ask that you please explain, with as much detail as possible, why you are unable to make your data available in one of the three places mentioned above so we can further assess whether or not this complies with PLOS’ Data Policy and determine how we can assist you in this matter.

**OUR RESPONSE:** As agreed with participants from the Whitehall II cohort study, ethics committee, and funders ([MRC Policy on Research Data Sharing](https://www.mrc.ac.uk/research/policies-and-guidance-for-researchers/data-sharing/)) data are shared for research purpose only. However, we are committed to maximizing the use of Whitehall II data to advance scientific knowledge, thus given the above mentioned restrictions, we welcome researchers to apply for data access (<https://www.ucl.ac.uk/epidemiology-health-care/research/epidemiology-and-public-health/research/whitehall-ii/data-sharing>).

ARTICLE FILE:

1. Please include the corresponding author's email address on the title page of your manuscript, indicated by an asterisk (*). Only an asterisk and the email address itself are required.

**OUR RESPONSE:** This is done.

2. Please remove author degree titles from the byline of your article file.

**OUR RESPONSE:** This has been removed.

3. Please ensure that all authors have at least one affiliation listed on the title page, and that all listed affiliations are attributed to at least one author. Affiliations should be listed in ascending numerical order in both the affiliation list and in the author byline.

**OUR RESPONSE:** This is done.

4. The name for your co-authors Mika Kivimäki and Séverine Sabia do not match across the article file and the submission form. Please ensure that all author names match verbatim, including the use of a middle name/initials and any special characters (accents, umlauts, etc.), as correct spelling and formatting of author names is vital for accurate indexing in PubMed.

**OUR RESPONSE:** Thank you, we have corrected the spelling of these names when necessary.

5. Please remove the funding information from your manuscript file. The published version is pulled automatically from the “Financial Disclosure” field in the submission form and must not be duplicated. Please therefore take this opportunity to check that the information in this field is correct and completed in full.

**OUR RESPONSE:** This is done.

6. Please remove the Competing Interests section from your manuscript file. The published version is pulled automatically from the "Competing Interests" field in the submission form and must not be duplicated. Please therefore take this opportunity to check that the information in this field is correct and completed in full.

**OUR RESPONSE:** Done as well.

7. Please remove your Author Contributions section from your manuscript file as this information is automatically pulled from the authors section of our submission form and must not be duplicated. Please therefore take this opportunity to check that the information in this field is correct and completed in full.

**OUR RESPONSE:** It has now been removed.

8. Please remove the data availability information from your manuscript file as this is automatically pulled from the "Data Availability" field in the submission form and must not be duplicated. Please therefore take this opportunity to check that the information in this field is correct and completed in full.

**OUR RESPONSE:** Now removed.

9. Please ensure that your Ethics statement is available in the Methods section of your manuscript in its entirety.

**OUR RESPONSE:** This appears on lines 119 to 121.

REFERENCES:

1. Please ensure your references are in the style of PLOS Medicine (ICMJE/Vancouver style). The reference style guide can be found here: <http://journals.plos.org/plosmedicine/s/submission-guidelines#loc-references>. You can also find a downloadable EndNote file in these guidelines to assist you. Please do a manual check as well, as the EndNote file does not catch everything. Please note that the references will be typeset as they appear so please ensure the format is consistent.

**OUR RESPONSE:** We have checked that the references are provided as requested.

2. Please put all reference citations in square brackets separated by either commas or dashes with no spaces, e.g. [1,2,3] or [1-3].

**OUR RESPONSE:** We have removed the spaces when necessary.

3. Please make sure all references are cited in ascending numerical order in the text.

**OUR RESPONSE:** This has been verified.

SUPPORTING INFORMATION:

1. Please ensure your supporting information files are individually uploaded to the File Inventory as file type 'Supporting Information' and are not present in the manuscript file.

**OUR RESPONSE:** Supporting files are uploaded as ‘Supporting Information’ and not present in the manuscript file.

2. Please upload your supporting information as individual files, and use the following naming format: S1 Text; S1 Fig; S1 Table, S1 Data, etc. Each file type should start as S1 and increase numerically: S1 Text, S2 Text, etc. Note that all relevant in-text citations and captions will need to be updated accordingly.

**OUR RESPONSE:** We have now modified the text as requested.

3. Please ensure that all Supporting Information files use the following format verbatim throughout the manuscript (including legends and all in-text citations): S1 Fig, S1 Table, S1 Text, S1 Data, etc. Each file type should start at 1 and increase numerically. Please note, however, that the file names themselves must use an underscore rather than a space, i.e. S1_Fig.tif, S1_Table.xlsx, S1_Text.doc, S1_Data.xlsx, S1_PRISMA_Checklist.docx, etc.

**OUR RESPONSE:** We have now modified the files names as requested.

4. Please ensure that each supporting information file has a legend at the end of the manuscript file, after the Reference list.

**OUR RESPONSE:** Legends have been added at the end of the manuscript.

5. Please note that your submission cannot contain any references to generic "supporting information" files and instead must refer to specific files using our naming convention (S1 Fig, S1 Table, S1 Text, S1 Data, SI PRISMA Checklist, etc.)

**OUR RESPONSE:** Thank you, we have removed reference to “supporting information” when it appeared and replaced as requested by specific file names.
